# Characterizing preferred terms for geographically distant simulations: distance, remote and telesimulation

**DOI:** 10.54531/dwti2869

**Published:** 2022-07-03

**Authors:** Todd P Chang, Rachel Elkin, Tehnaz P Boyle, Akira Nishisaki, Barbara Walsh, Doreen Benary, Marc Auerbach, Cheryl Camacho, Aaron Calhoun, Stephanie N Stapleton, Travis Whitfill, Trish Wood, Jabeen Fayyaz, Isabel T Gross, Anita A Thomas

**Affiliations:** 1 Division of Emergency Medicine & Transport, Children’s Hospital Los Angeles/Keck School of Medicine, University of Southern California, Los Angeles, CA, USA; 2 Division of Pediatric Emergency Medicine, New York-Presbyterian Morgan Stanley Children’s Hospital-Columbia University Irving Medical Center/Columbia University Vagelos College of Physicians and Surgeons, New York, NY, USA; 3 Division of Pediatric Emergency Medicine, Boston Medical Center/Boston University School of Medicine, Boston University, Boston, MA, USA; 4 Division of Pediatric Critical Care, Children’s Hospital of Philadelphia, University of Pennsylvania, Philadelphia, PA, USA; 5 Division of Emergency Medicine, Boston Children’s Hospital, Harvard University, Boston, MA, USA; 6 Division of Pediatric Emergency Medicine, NYU Langone Medical Center, New York University, New York, NY, USA; 7 Departments of Pediatrics and Emergency Medicine, Yale University School of Medicine, New Haven, CT, USA; 8 Simulation and Outreach Education, Nationwide Children’s Hospital, Columbus, OH, USA; 9 Division of Critical Care, Norton Children’s Hospital, University of Louisville, Louisville, KY, USA; 10 Department of Emergency Medicine, Boston Medical Center/Boston University School of Medicine, Boston, MA, USA; 11 Starship Child Health, Auckland, New Zealand; 12 Division of Emergency Medicine, Hospital for Sick Children, University of Toronto, Toronto, ON, Canada; 13 Division of Pediatric Emergency Medicine, Yale University School of Medicine, New Haven, CT, USA; 14 Department of Pediatrics, Division of Emergency Medicine, University of Washington School of Medicine, Seattle Children’s Hospital, Seattle, WA, USA

## Abstract

**Background:**

Simulationists lack standard terms to describe new practices accommodating pandemic restrictions. A standard language around these new simulation practices allows ease of communication among simulationists in various settings.

**Methods:**

We explored consensus terminology for simulation accommodating geographic separation of participants, facilitators or equipment. We used an iterative process with participants of two simulation conferences, with small groups and survey ranking.

**Results:**

Small groups (n = 121) and survey ranking (n = 54) were used with *distance, remote, and telesimulation* as leading terms. Each was favored by a third of the participants without consensus.

**Conclusion:**

This research has deepened our understanding of how simulationists interpret this terminology, including the derived themes: (1) physical distance/separation, (2) overarching nature of the term and (3) implications from existing terms. We further deepen the conceptual discussion on healthcare simulation aligned with the search of the terminologies. We propose there are nuances that prevent an early consensus recommendation. A taxonomy of descriptors specifying the conduct of *distance, remote* and *telesimulation* is preferred.

## Introduction

As healthcare simulation evolved to accommodate public health regulations around social distancing and work-from-home mandates, the ways in which simulations are conducted have changed substantially in many institutions. Simulationists have adapted their curricula, environments and technological framework to accommodate simulations in which learners and facilitators may not all be in the same room [1]. There is a broad consensus that new techniques or technologies are required to conduct effective simulation that accommodates geographical separation of participants, facilitators and equipment [2]. However, the solutions have varied in their approaches. Hybrid instructional approaches utilizing more than one modality or instructional design method are common [3]. The approach in how different centers, instructors and simulation operators have conducted this type of simulation is quite variable.

Duff et al. describe simulation at a distance as ‘a novel offshoot of an established discipline (simulation-based education)’ [4]. Disciplines evolve more and mature through the development of shared frameworks and vocabulary, and then through applications from in-person simulations and consolidation of knowledge and evidence [5]. Duff et al. also proposed that we are at the edge of a precipice at which a ‘development of new vocabulary to describe the new innovations and provide further clarity of definitions’ begins [4]. In fact, the Society of Simulation in Healthcare (SSH) Dictionary added a 2020 addendum to define new terms in the literature; these included a variety of terms, including *remote simulation, virtual simulation, distance simulation, online simulation* and *telesimulation* [6]. Simulationists in multiple parts of the world anecdotally had distinct impressions, assumptions and preferences for specific nomenclature to describe simulation at a distance.

Therefore, multiple simulation societies met in an effort to formulate a common understanding of the various terminologies describing simulation at a distance and whether a singular nomenclature would be possible.

We present the findings in two parts. First, the results of a large-scale multi-society exercise to determine consensus terminology for geographically distanced simulation activities are presented. We emphasize simulationists’ preferences for, and aversions to, potential terms. Second, the results are contextualized through how the three major adjectives, *distance, remote* and *tele*-, are used and interpreted in disciplines outside of healthcare simulation.

## Terminology consensus exercises (2020)

In the Fall of 2020, We conducted a multistep consensus process using a parallel quantitative and qualitative content analysis design [7] to describe the overall preferences and justifications for terms that best encompasses all simulations when people are *not* in the same room (i.e. physical or geographical separation). Any wholly digital simulation (e.g. a multi-player serious game) was excluded. We began with an overarching purpose to come to a consensus on the optimal term for simulation with geographical separation. Of note, we consider the terms geographical separation and physical separation to mean the same thing.

**Figure 1 :**
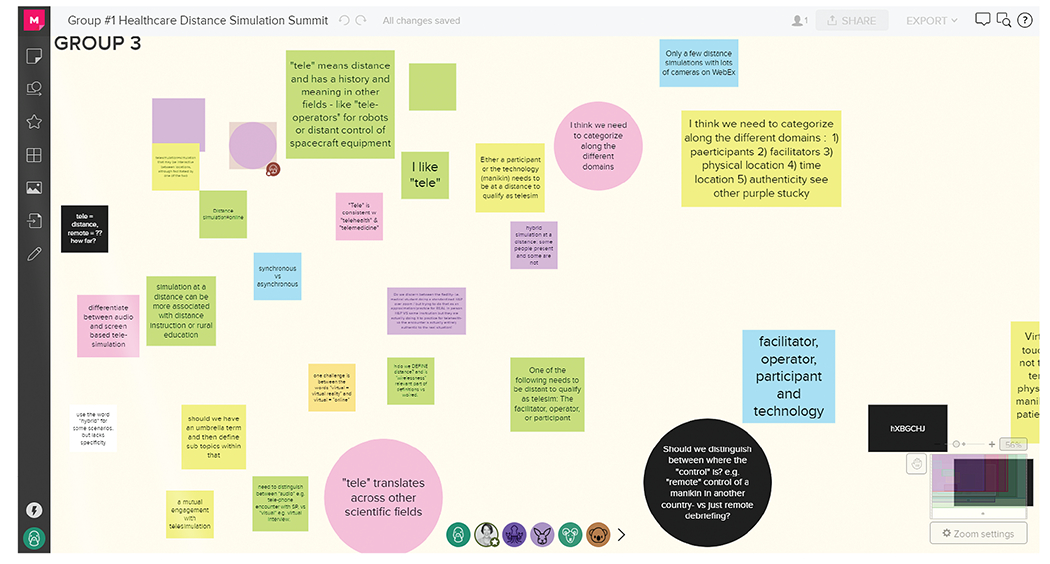
Group discussion board. Round 1 group exercises using MURAL (Tactivos, Inc., San Francisco, CA) at the virtual *Healthcare Distance Simulation Summit* in August 2020.

**Figure 2 :**
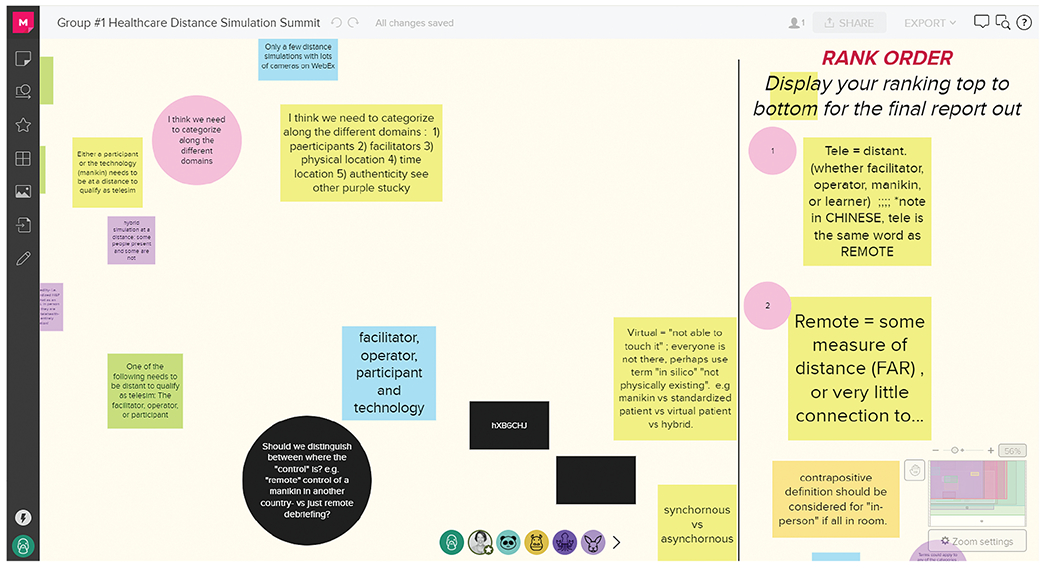
Group discussion board with ranking. Round 1 group exercises with ranking using MURAL (Tactivos, Inc., San Francisco, CA) at the virtual *Healthcare Distance Simulation Summit* in August 2020.

The exercises included members from the International Network for Simulation-based Pediatric Innovation, Research, and Education (INSPIRE), International Pediatric Simulation Society (IPSS), Netwerk KinderSimulation (NKS) and the Pediatric Simulation Training and Research Society (PediSTARS) in India. This was a convenience sample of simulation networks. Conference attendees represented one or more of these simulation organizations, and we attempted to include an international group to obtain a diverse perspective with the goal of an English-language consensus. Small group ranking exercises were conducted as part of the *Healthcare Distance Simulation Summit* on August 14, 2020, using a virtual note board application MURAL (Tactivos, Inc., San Francisco, CA), as noted in Figure 1 and Figure 2 group discussion boards. Then, an individual follow-up survey was administered to another set of participants attending the INSPIRE at IPSSV (International Pediatric Simulation Symposia – Virtual) meeting on October 29, 2020. All terms used in the SSH Dictionary update [6] were introduced as examples in alphabetical order: *distance simulation, remote simulation, telesimulation and virtual simulation*. These terms were specifically highlighted because their prevalence required an update to the SSH Dictionary. A consultation with a semantics professor outside of the healthcare simulation discipline then confirmed that they were, in fact, all ‘orthogonal’ in the linguistic sense, defined as no obvious inherent bias in the terminologies. We used a content analysis framework [7,8] for both parts of the consensus exercises to derive themes, with member checking to improve the trustworthiness of this approach [9]. The quantitative portions of the survey – i.e. ranks of preferred terms – were analyzed using descriptive statistics using SPSS version 27 (IBM, Chicago, IL). This study received Institutional Review Board (IRB) exemption from Children’s Hospital Los Angeles.

## A lack of consensus, but emerging nuances

A total of 121 simulationists in August 2020 and 54 in the October 2020 survey participated. While the exercises yielded no single consensus term, there were three leading contenders. In alphabetical order, the three preferred terms were *distance simulation, remote simulation* and *telesimulation*. Table 1 summarizes round 1 findings, and Table 2 summarizes the overall rankings from round 2.

## Simulationists’ perspectives on terms

We discovered three overarching themes from the consensus exercises that underpinned the groups’ justification for the rankings: 1) Physical Distance and Separation, 2) Overarching Nature of the Term and 3) Implications from the Existing Term. Full results are available in Appendix.

### Physical Distance and Separation –

This theme was repeated in several iterations across many terms. For many participants, the term was required to convey the sense of physical or geographical distance where telecommunication technology was required to connect participants and/or facilitators. Opinions were divided on the optimal term that represented the geographical distance:

- ‘Tele – everyone is outside the room; definition over a distance’
- ‘*Distance* encompasses the group being physically distanced from each other’
- ‘*Distance* simulation doesn’t fully suggest that people are NOT in the same room as this could imply a 6-foot (2 m) rule for COVID-19 precautions rather than *remote* learning’
- ‘I think *remote* accurately conveys that everyone is far from one another but makes it clear that this is not purely virtual’
- ‘*Remote* feels too ‘distant,’ this doesn’t need to be miles and miles away’

### Overarching Nature of Term –

This was a very prominent theme expressed among all groups. Simulationists struggled with the conflicting desire for a term with adequate specificity versus broad inclusivity. Among those desiring specificity, existing terms lacked information on *temporal* aspects and technology requirements to successfully conduct the simulation. These resulted in conversations about synchrony or asynchrony, and whether a term encompassed both or not:

- ‘Virtual: Asynchronous is included, or synchrony is not assured’
- ‘Distance: Asynchronous implication – passive’

Some participants found the term *distance* too vague or non-specific to suggest the exact nature of how participants were separated in simulation and ranked the term poorly. Within the term *tele*-, there may be implied technology requirements:

- ‘*Distance* is not a precise enough term (how much distance – one room over?)’
- ‘I interpret *remote* as more general than *distance;* allows for more flexibility’
- ‘I think the semantics of the term “tele-” is more inclusive of the simulation environment. It implies that the simulation is being performed as a typical simulation, just over tele-communications’

Terminologies were also criticized or favored depending on whether they invoked required technologies (e.g. teleconferencing software):

- ‘How do we define “distance”?’ and is ‘wirelessness’ [a] relevant part of definition vs wired?’ [sic]
- ‘*Tele*- brings to mind a very specific vision of using Zoom(R) and being very distant’

Concerns about the use of *Telesimulation* centered on specifying whether the object of the simulation was to replicate in-person care or telehealth / telemedicine:

- ‘Tele: In [an Objective Standardized Clinical Examination] (OSCE), tele-medicine OSCE is different from remote OSCE (which evaluates the actual provider-patient encounter)’

Finally, further specificity arguments asked for clarity regarding the participants and facilitators, and their interactions. *Hybrid simulation* was a term that encompassed many types of geographical distribution patterns:

- ‘One of the following needs to be distant to qualify as telesim: The facilitator, operator or participant’
- ‘I think we need to categorize along the different domains: 1) participants 2) facilitators 3) physical location 4) time location and 5) authenticity’

### Implications from the Existing Term –

The final prominent theme included rationales based on what the English word evoked – from connotations to related, associated terms. This was the principal theme that suggested *virtual simulation* would not be favored as a term:

- ‘Distance: Close to distance learning; joining campuses’
- ‘Virtual - seems to imply a virtual reality headset’
- ‘Remote: Smaller cities; resource limited; rural’
- ‘Tele’ is consistent with ‘telehealth’ and ‘telemedicine’

Unrelated terms that share the same word roots affected how participants considered the terms such as *distance, remote* or *telesimulation*:

- ‘It makes me think about *distance* learning - a term that is used for online school programs’
- ‘As a *remote*-controlled car or helicopter; the [simulation] is also done *remotely’*
- ‘[There is] familiarity with *tele*- used for other health-related activities’

These associations also led to connotations and implications of isolation, negative emotions or, particularly for *telesimulation*, connections with clinical care delivery:

- ‘*Distance* has a stronger connotation of not being “together” than the rest for me. We are already isolated enough these days!’
- ‘*Remote* does not sound very warm!’
- ‘*Tele*- is linked to service and not education’

## Conceptual analysis of healthcare simulation

We present results from two successive exercises designed to explore unifying terminology for simulations with geographical distancing. Simulationists did not reach a consensus on any of the three terms supported by the SSH Healthcare Simulation Dictionary [6]. Each term was equally preferred in the English language: *distance simulation, remote simulation* and *telesimulation* for a variety of reasons. The pandemic forced a very rapid change in healthcare and healthcare simulation practice, and based on our work, it is too early to crystallize into a single terminology. Here, we turn to the considerations that simulationists echoed about the three terms and look towards other disciplines with similar terminology.

Healthcare simulation roots itself on maintaining some level of fidelity to the actual practice of healthcare, whether one-on-one with a patient or among a team surrounding a critical or operative patient. Various aspects of fidelity provide a framework for optimal simulations for learning and are predicated on the idea that clinical care is typically done with in-person teams. Nuances of communication, tactile feedback, situational awareness and even the sense of autonomy and control are played out in a safe learning environment during simulation. The science of simulation does not propose that perfect fidelity is required for optimal learning transfer and experiences. Rather, breaking from perfect fidelity can be both helpful and harmful to learning transfer. The ability to pause a scenario enables reflection-in-action [10]. The ability to remove sensory inputs enables a lower cognitive load for novices [11]. Poor fidelity may conversely lead to learners acquiring incorrect skills or make assumptions about the patient situation.

One could then argue that the COVID-19 pandemic has not only changed the assumptions of healthcare and healthcare simulation in many locales. In considering the urge for new terms, we reflect on the idea that geographical distancing during simulations is a necessary variation in the ‘prototypical’ mannequin-based simulation secondary to pandemic-mandated physical distancing. Cognitive linguistics explains that new terminologies emerge as subcategories from the prototype require greater specificity [12]. Furthermore, scientific terminologies depend substantially on perspective and expertise, rather than a neutral or absolute definition [12]. In our example, we consider two potential perspectives within healthcare simulation with geographical distance: from within healthcare practice, and outside of healthcare.

## Tele- and remote in healthcare practice

Telesimulation is a direct descendent of *telehealth* and its predecessor *telemedicine.* Bashshur proposed that a ‘common thread in all definitions of telemedicine (literally, medicine at a distance) to date is the geographic separation between two or more interactants engaged in healthcare’ [13]. The expansion to *telehealth* was a ‘more inclusive concept’ introduced in 1978 as a more systems-level activity. Without this upgrade in terminology, ‘these definitions limit the purview of telemedicine to *remote* patient care’ [emphasis added] [13]. In 1995, Telemedicine first appeared in a scholarly publication journal, entitled as its namesake, *Telemedicine* [13]. The strong link of *telesimulation* to healthcare as a system of practice was echoed in our findings.

*Remote*, in the healthcare world, has been used in terms like *remote monitoring.* Remote monitoring offers observation and data collection that transcends distance but primarily concerns itself with the technological ability to monitor data. Remote vital signs monitoring, for example, is featured in engineering literature through its innovative technological capabilities [14]. *Remote* is often used in electronic communications of smart objects and is the preferred term for current digital innovations in the electronics consumer world [15]. *Remote control* also points to a technological perspective on how to interact. It is likely that simulationists preferring the term *remote* gravitate towards the technological complexity of conducting simulations across distances, with less focus on the social isolation or systems-level considerations of *distance* and *telesimulation.*

Finally, one term was unanimously voted out. Semantic similarity for *virtual simulation* to *virtual reality* contributed to this term’s removal before the 2nd survey; the term *virtual*, as of this writing, evokes the relatively novel technology of virtual reality and the typically marketing-heavy term of ‘virtual meeting’ that is too specific to a technological modality. While *virtual simulation* fell out of favor because of the implications of the word *virtual*, still others felt that *distance, remote* and *tele*- were reasonably inclusive and could use some modifiers.

## Limitations

Simulation networks that participated were a convenience sample, and there is potential for selection bias in that main and byline authors had connections to these networks. We included international simulation societies to obtain a broader linguistic perspective, although this consensus was limited to the English language. Virtual meetings were scheduled via online scheduling polls to accommodate international schedules, although it was impossible to schedule all meetings during daytime business hours for all parties involved given global time differences. Authors put forth an effort to have varied meeting times to be more inclusive of international time zone. Attendees were healthcare simulationists – primarily physicians and nurses who are simulation leaders, but some participants represented other aspects of health care such as medical students or respiratory therapists. This elucidates a limitation of the attendees in that while many simulation experts are in physician and nursing roles, there are others in various other aspects of health care; thus, a broader perspective of simulationists with varying primary professions could have been pursued. We acknowledge that our failure to include authorship from outside of the United States or Canadian is a limitation, and that this may have affected the analysis. Future projects could more actively engage international colleagues beyond the United States and Canada and be more language inclusive.

## Distance, tele- and remote outside of healthcare simulation

Thought leaders in other disciplines echo concepts and considerations from simulationists on how the terms *distance, tele*- and *remote* are used in their fields.

Concerns over the term *distance* are seen in multiple disciplines for its implication of loneliness or being different. In theology, educators worried about the discordance of the term *distance* and spiritual formation wherein the ‘material absence of physical presence in collective worship was striking’ [16]. In engineering, distance education was proposed as a win for women, who could access traditionally male-dominated fields, courses or institutions while balancing home or childcare responsibilities and less in-person harassment. However, there is still a notion of being second-class, inferior, ‘or [a] compromise – for women with children and without much free time’ [17]. In engineering education, for example, there is a simple problem of unequal access to on-campus university laboratories.

The concept of *distance, remote* and *tele*- as terms used in higher education generated similar discussions with results comparable to our findings in simulation, starting in the 19th century. The higher education community also used multiple terms without consensus until 1982, when the term *distance education* was finalized at an International Council for Correspondence Education conference for Higher Education [18]. Some educators were dissatisfied with the forceful implication of geographical separation and wished to emphasize instead the more philosophical paradigm changes, in which agency for learning moves away from a university institution to the learner [18]. Others wanted more technology emphases, leading to terms such as *virtual* and *tele* added to distance education. Furthermore, the idea that knowledge is democratized and accessible led to further terms such as *distributed learning* [19].

## Conclusion

At present, simulationists do not have a full consensus on a single overarching terminology for simulations conducted where the participants or facilitators are geographically separated. The three most favored terms are (alphabetically): *distance, remote* and *telesimulation.* Based on our deepened understanding, it is likely that these three terms will evolve to differentiate each other over time, and a more comprehensive description, rather than a simple term, is necessary for this evolving modality of simulation.

## Figures and Tables

**Table 1:** Rank order of nomenclature during the round 1 group exercise

Rank	Group 01	Group 02 (German group)	Group 03	Group 04	Group 05	Group 06	Group 07	Group 08	Group 09	Group 10
Best	Distance	*Fern*-	Tele-	Tele-	Adaptive	Tele-	Remote	Remote	Meta-	Interactive Distance
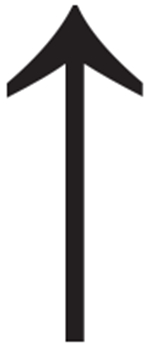	Remote	*Tele*-	Remote	Distance	Online	Distance	Distance	Tele-	Remote	Interactive Remote
		*Virtuelle*			Tele-	Remote	Tele-	Distance	Distance	Distance
		*Digitale*			Distance	Virtual		Virtual		Remote
		*E*-			Remote	E-				
Worst					Virtual					

A summary of overall rankings from round 1 group exercises using MURAL (Tactivos, Inc., San Francisco, CA) at the *Healthcare Distance Simulation Summit* in August 2020.

**Table 2:** Rank order of the 3 terms: *distance, remote and telesimulation*

Rank	Distance	Remote	Tele-
#1	14 (28%)	17 (34%)	19 (38%)
#2	20 (40%)	16 (32%)	14 (28%)
#3	16 (32%)	17 (34%)	17 (34%)

A summary of overall rankings from round 2 follow-up survey to participants attending International Network for Simulation-based Pediatric Innovation, Research, and Education (INSPIRE) at International Pediatric Simulation Symposia – Virtual (IPSSV) meeting on October 29, 2020.

## Data Availability

None.

## What this study adds?

- Healthcare simulation with geographic separation is more common with social distancing requirements related to the COVID-19 pandemic with unclear consensus on terminology
- Consensus exercises on terms related to healthcare simulation at a geographic distance found the terms distance, remote and telesimulation to be preferred

## APPENDIX. RESULTS FROM THE FIRST EXERCISE AND SURVEY COMMENTS

**Table:**

Theme	Category	Round 01 Quotations
Physical Distance and Separation	Representation of Geographical Distance	• ‘Tele - everyone is outside the room; definition over a distance’ • ‘Tele = distance; remote =?? how far?’ • ‘Contrapositive definition should be considered for “in-person” if all in room’ • ‘Distance implies physical separation; learners are physically apart (two different locations)’ • ‘Remote: means distance (different places); implies far from a central location; really far’
	Representation of Pandemic-related Separation	• ‘Remote: “working from home”’ • ‘Given the pandemic trigger, “distance” associates what we are doing closely with it. This may be good or bad depending on the situation’
Overarching Nature of the Term	Temporality - whether asynchrony or synchrony is implied	• ‘Geographically vs Time separation / Not talking about Asynchronous / Solo; Time - synchronous / asynchronous’ • ‘Could use synchronous or asynchronous adjacent to the agreed-upon “umbrella term” to capture time aspect’ • ‘Online: Most likely to be asynchronous’ • ‘Distance: Asynchronous implication – passive’ • ‘Remote: Only Space, doesn’t cover time’ • ‘Need something to encompass geography and time’ • ‘Virtual: Asynchronous is included, or synchrony is not assured’ • ‘Is it really possible to deal with asynchronous sims in this nomenclature? We have doubts that group learning can occur if synchrony is not there. May be time separation dependent though’ • ‘Interactive Social fits all spatial categories, but can you be social asynchronously? Or is there a minim[um] synchrony threshold?’
	Implied Technology Requirement	• ‘Distance sim can happen with a space if participants are separated with partitions or social distancing – allows for low tech solutions’ • ‘Tele: Restricts to the technology / TELEphone/camera/audio / anchored in the technology using’ • ‘How do we define “distance”?’ and is ‘wirelessness’ relevant part of definitions vs wired?’
	Tele- for Telehealth or Telemedicine vs In-person care delivered through Telesimulation	• ‘Tele: In [an Objective Standardized Clinical Exam], tele medicine OSCE is different from remote OSCE (which evaluates the actual provider-patient encounter)’ • ‘Tele: For students, it may imply tele-medicine training’ • ‘Telesim suggests a simulated telehealth encounter (could be a great context for sim! But is not inclusive of every type of sim we are thinking about today)’ • ‘Tele: Using when using telehealth equipment’ • ‘Do we discern between fidelity - i.e. med student doing a standardized [History & Physical Exam] over Zoom but trying to do that as an approximation practice for REAL in person H&P…vs…same interaction but they are actually doing it to practice for telehealth, so the encounter is actually entirely authentic to the real situation?’
	Clarity regarding participants, facilitators and their interactions	• ‘Either a participant or the technology ([mannequin]) needs to be at a distance to qualify as telesim’ • ‘Hybrid simulation at a distance; some people present and some are not’ • ‘One of the following needs to be distant to qualify as telesim: The facilitator, operator or participant’ • ‘I think we need to categorize along the different domains: 1) participants, 2) facilitators, 3) physical location, 4) time location and 5) authenticity’ • ‘Telesim: that learner and facilitator in different spaces’ • ‘Distance: means learners are physically apart (2 different locations)’ • ‘Important to be clear some training are on-site, while some training utilize hybrid in learners’ location’ • ‘From a psychological safety standpoint, it is important to notify learners and facilitators about the location and [whether it is] synchronous / asynchronous’
**Implications from the Existing Term**		• ‘Distance: Close to distance learning; joining campuses’ • ‘Distance simulation vs. Distance education’ • ‘Virtual - seems to imply a virtual reality headset’ • ‘One challenge is between the words Virtual = Virtual Reality and Virtual = Online’ • ‘Virtual – 2D Virtual Reality and 3D Virtual Reality’ • ‘Virtual: Too similar to Virtual Reality; but has been used in “virtual table top”‘ • ‘Virtual: More gaming, like a movie – feels less educational’ • ‘Virtual OSCE is an officially used term – just meaning that it is not physical but it is done online’ • ‘Remote: Smaller cities; resource limited; rural’ • ‘Tele’ is consistent with ‘telehealth’ and ‘telemedicine’ • ‘Tele’ means distance and has a history and meaning in other fields – like ‘tele-operators’ for robots or distant control of spacecraft equipment’ • ‘Tele: Restricts to the technology / telephone/camera/audio, anchored in the technology used’ • ‘Tele: Cloud-based technical solutions’ • ‘Tele: For students, it may imply tele-medicine training’ • ‘Online: Not expecting physical components – less comprehensive’ • ‘e-Sim: e for electronic; internet based; linked with technology’

**Table:**

Round 02 – Selected Survey Comments	Distance Simulation	Remote Simulation	Telesimulation
(+) Physical Distance and Separation – emphasizes distance	‘Best description that doesn’t imply you are alone’ ‘Distance encompasses the group being physically distanced from each other’ ‘This emphasizes the geographical distance’ ‘This is probably an accurate translation of what is happening - the presence of physical distance between facilitator and learners or among learners in a hybrid model’	‘I believe remote simulation represents any distance you could be…’ ‘Remote to me signifies away and not in the same room’	
(+) Overarching Nature of Term – encompassing, implies technology, temporality	‘Encompasses different and diverse approaches but not all’ ‘I feel that it is a basic definition that learners and facilitators are distance[d] from each other regardless of what technology they use’ ‘It implies some distance without dictating amount [of distance]’ ‘This word is also nice since it covers the hybrid model’ ‘This seems to encompass the most ways that simulation could be occurring’	‘…requiring some kind of tech to bring people into the same space on a virtual or remote setting’ ‘Seems the most encompassing for simulations that utilize VR from different locations’	‘Encompasses the teleconferencing (or videoconferencing) that is incorporated into the simulation’ ‘I think the semantics of the term “tele-” is more inclusive of the simulation environment. It implies that the simulation is being performed as a typical simulation, just over tele-communications’ ‘I believe that most people know that “tele” implies physical separation and a connection via screen (computer, robot, etc.) that’s electronically facilitated’ ‘You may be next door, which is not remote or distant from each other’ ‘Telesim, in my mind, is the best fit as many of us understand that tele relates to using audio and visual tech to do something from another location and this is what I understand telesim to be’
(+) Implication from Existing Terms – positive notions of familiarity		‘Similar to “Remote Teaching”’ ‘As Remote control car, or helicopter this is also done remotely’	‘Familiarity with tele-, used for other health-related activities’ ‘I think all of the terms have pros and cons. I think the only value for tele-sim is the recognition of those outside our field: telehealth, telepresence, tele-sim’ ‘Tele seems to connect with my native word for phone – *telefons*’
(−) Physical Distance and Separation – not precise enough description of distance	‘Distance could mean some but not all members are distanced’ ‘Distance simulation doesn’t fully suggest that people are NOT in the same room as this could imply a 6-foot rule for COVID-19 precautions rather than remote learning’ ‘Distance may be interpreted as either remote or in the same room but farther apart than normal’ ‘Distance is not a precise enough term (how much distance, one room over)?’ ‘It has a stronger connotation of not being together than the rest [of the terms] for me. We are already isolated enough these days!’	‘Remote, to me, gives a [sense of] long distance away, which may or may not be true’ ‘Remote feels too “distant” – this doesn’t need to be miles and miles away’	
(−) Overarching Nature of Term – does not clearly convey delivery, relies on technology	‘This term is not intuitive to me when I hear it’ ‘Didn’t seem to capture the definition as well’ ‘It does not convey the challenge adequately’	‘Also suggests remote (control) of events as a required facet’ ‘I think the term remote implies that there is a barrier to the simulation session. It must be performed separated from the simulation materials by either time or space’ ‘Remote has a somewhat [negative] connotation for me. Who is remote and who is central?’	‘Because [tele] refers to technique’ ‘Tele-sim brings to mind for me a telehealth visit (one person talking via video to one other person or a group). There are a lot of models for distance sim occurring, so this term seems limiting’ ‘Tele- feels a bit more tied to a specific technology/style of encounter (a la telemedicine), so feels less flexible to me than the other terms’ ‘This brings to mind a very specific vision of using Zoom and being very distant’
(−) Implication from Existing Terms – negative notions of familiarity	‘It makes me think about distance learning – term that is used for online school programmes’	‘For some people, remote means ‘rural’ – resource limited. This is an implicit bias some may have’ ‘Remote does not sound very warm!’	‘I think of phone or telehealth’ ‘[It] also sounds more dated – I can imagine this being of limited value in the future. Let’s not go through this again in 15 years!’ ‘[It] will get outdated fast’ ‘I think telesimulation is confusing with telehealth’ ‘[Tele] is linked to service and not education’ ‘Reminds me too much of telehealth and television’

Results from exercise and survey comments. On the left are the three final themes and representative quotes by category.
